# Glutamate-Sodium Discrimination Status in Adults Is Associated with Salt Recognition Threshold and Habitual Intake of Discretionary Food and Meat: A Cross-Sectional Study

**DOI:** 10.3390/ijerph191711101

**Published:** 2022-09-05

**Authors:** Isabella Hartley, Andrew Costanzo, Djin Gie Liem, Russell Keast

**Affiliations:** CASS Food Research Centre, School of Exercise and Nutrition Sciences, Deakin University, Victoria 3125, Australia

**Keywords:** taste, diet, umami, psychophysics, monosodium glutamate, sodium, salt

## Abstract

Umami non-discriminators (NDs) are a sub-group of the population with a reduced ability to discriminate between monosodium glutamate (MSG) and sodium chloride (NaCl) compared to umami discriminators (UDs). No research has investigated umami and salty taste perception associations across detection threshold (DT), recognition threshold (RT), and suprathreshold intensity perception (ST) or the habitual dietary intake of ND. Adults (*n* = 61, mean age of 30 ± 8 years, *n* = 40 females) completed taste assessments measuring their DT, RT, and ST for salty, umami (MSG and monopotassium glutamate (MPG)), and sweet tastes. To determine the umami discrimination status, participants completed 24 triangle tests containing 29 mM NaCl and 29 mM MSG, and those with ≥13 correct identifications were considered UDs. Habitual dietary intake was recorded via a food frequency questionnaire. NDs made up 14.8% (*n* = 9) of the study population, and UDs made up 85.2% (*n* = 52). NDs were less sensitive to salt at RT (mean step difference: −1.58, *p* = 0.03), and they consumed more servings of meat and poultry daily (1.3 vs. 0.6 serves, *p* = 0.006); fewer servings of discretionary food (1.6 vs. 2.4, *p* = 0.001); and, of these, fewer salty discretionary foods (0.9 vs. 1.3, *p* = 0.003) than NDs. Identifying these NDs may provide insight into a population at risk of the overconsumption of discretionary foods and reduced intake of protein-rich meat foods.

## 1. Introduction

Umami taste, elicited predominately via the sodium salt of L-glutamate (monosodium glutamate (MSG)) in psychophysical research, is described as a savory, brothy, and meaty taste [1]. Since the discovery of an L-glutamate taste receptor, T1R3 [2], umami has been commonly labelled as the fifth basic taste. Recent discussions on classification criteria of basic tastes suggest that based on a reduced perceptual salience in comparison to the traditional four basic tastes, umami (and other tastes) may occupy a potential sub-group of tastes [3,4]. This sub-group has been suggestively termed ‘alimentary tastes’ [4]. Alimentary tastes are proposed to be important for post-ingestive signaling throughout the alimentary tract and for modulating ingestive behaviors, rather than for strong perceptual salience [4,5,6]. The importance of umami as an alimentary taste has been illustrated in regard to its role in food-intake control [7], increasing post-ingestive satiety [8] and energy compensation [9].

Psychophysical research has investigated individual differences in umami taste perception, and a standard methodology has been developed to identify a sub-group of the population with a reduced ability to discriminate L-glutamate from the sodium ion in MSG [10,11,12]. This methodology involves participants completing a series of triangle tests containing isomolar (29 mM) MSG and NaCl solutions [10,12]. For consistent categorization of the umami discrimination status, conducting 24 triangle tests is optimal; if participants can correctly identify the odd sample in ≥13/24 tests, they are considered an umami discriminator (UD) [12]. The sub-group with an inability to discriminate between 29 mM MSG and NaCl has been termed umami non-discriminators (NDs) [12,13,14]. NDs are likely to predominately be perceiving the sodium ion in MSG, rather than L-glutamate [10]. Considering that the discrimination task contains both NaCl and MSG, it may provide information regarding the NDs’ salty taste. It has been reported that NDs perceive more saltiness and less savoriness in MSG [13], perceive lower suprathreshold (ST) taste intensity from monopotassium glutamate (MPG), and have a lower MSG detection threshold (DT) than UDs [11]. This indicates that they have a reduced ability to perceive L-glutamate. However, when evaluating broths that contain sodium in isolation of glutamate (i.e., NaCl) compared to broths with sodium (NaCl) plus MSG, NDs have a reduced taste intensity for both broths compared to UDs, indicating that the perception of both L-glutamate and sodium may be attenuated in NDs [14]. This is further supported by findings that NDs perceive less intensity for salt ST solutions than UDs [14]. Finally, NDs may also perceive less sweet taste intensity [13] and consume more sugar and fried potatoes and less seaweed than UDs [15]. The association with the sweet taste is interesting because sugars and glutamate share the receptor subunit T1R3 [16].

Considering that the prototypical umami stimuli (MSG) contains a sodium ion, it is pertinent to investigate whether NDs have an attenuated taste function to L-glutamate or whether their salt taste function is influencing their MSG taste perception. Whether NDs and UDs show variation in taste perception for non-sodium L-glutamate tastants, such as MPG, across multiple taste dimensions has not been investigated. Moreover, research is yet to evaluate differences in the taste perception in NDs for salty and sweet tastes across different taste dimensions (DT, recognition threshold (RT), and ST). Identifying any associations that may exist is important for developing an understanding of inter-individual variation in umami taste perception and of whether sensitivity to sodium is playing a role in the discrimination task. Finally, limited research has investigated whether NDs have a different dietary intake to UDs. Considering the importance of umami as an alimentary taste and its role in the regulation of food intake and post-ingestive signaling, it is essential to understand umami taste perception and behavioral outcomes, particularly in umami NDs.

## 2. Aim

The primary objective was to compare taste perception, including umami (MSG and MPG), salty, and sweet tastes across multiple taste dimensions (DT, RT, and ST). A secondary objective was to assess differences in the habitual dietary intake between UDs and NDs.

## 3. Materials and Methods

### 3.1. Overview

As part of a larger intervention study, participants attended the CASS Food Research Centre, Deakin University, for their baseline taste assessments sessions. The ethics of the study were approved by the Deakin University Human Ethics Advisory Group Health (HEAG-H 094-2019), and participant consent was obtained according to the Declaration of Helsinki. A sample size calculation was performed in G*Power based on pilot data, which suggested that a total sample size of *n* = 56 would detect a difference in the salt RT between UDs and NDs, with an effect size of d = 0.78 and α = 0.05, with a power of 1-β = 0.80 [17].

**Figure 1:**
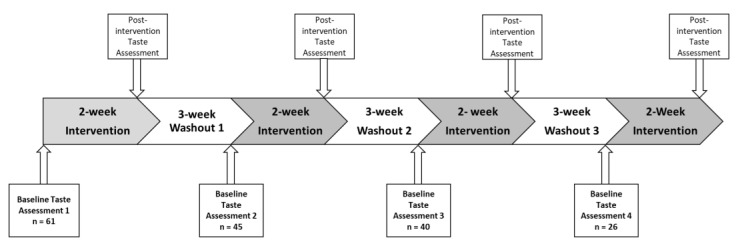
Outline of the larger intervention study; this study explored the baseline taste assessments prior to each intervention arm, and the total number of participants that completed each baseline taste assessment is outlined. All taste outcome measures were assessed at each repeat baseline taste assessment, including the discrimination status assessment, detection threshold, recognition threshold, and suprathreshold for salt, monosodium glutamate (MSG), monopotassium glutamate (MPG), and sucrose.

To be eligible to participate, participants must have been between the ages of 18–50-years-old, non-smokers, without food allergies, not following a specific diet, and not currently pregnant. The number of taste assessment sessions participants attended ranged from one to four; this varied due to participants completing a different number of intervention treatments in the larger study, thus attending a different number of repeat baseline taste assessment sessions. Data collection for the larger study was required to end due to mandatory laboratory closures, resulting in the varied number of taste assessment sessions participants completed; see Figure 1 for the larger study timeline overview.

In total, *n* = 61 participants completed the study, and of these, *n* = 16 (26%) participants attended one session, *n* = 5 (8%) attended two, *n* = 14 (23%) attended three, and *n* = 26 (43%) attended four repeated taste assessment sessions; during each taste assessment session, the same taste measures were taken. All taste assessment sessions completed by each participant were included in the final analysis. All taste assessment sessions occurred at 8.30 a.m., and participants were instructed to not eat or drink anything (excepting water) or to brush their teeth a minimum of one hour prior to the session commencement.

All tasting was conducted under red lights to prevent any visual differences influencing the results, with participants wearing nose clips to isolate taste. All solutions were presented with randomized, unique 3-digit blinding codes. Concentrations for the various psychophysical tests were prepared using filtered water. Tastants included sucrose for the sweet taste (Woolworths group, Baulkham Hills, NSW, Australia), sodium chloride for the salty taste (Saxa, Maryvale, NSW, Australia), and MSG (Ajinomoto Corporation, Tokyo, Japan) and MPG (Ajinomoto Corporation, Tokyo, Japan) for the umami tastes.

### 3.2. Anthropometrics

The participants’ height (cm) (Seca 213 Portable stadiometer, Hamburg, Germany) and weight (kg) (Body Scan Composition Monitor Scale, Tanita, Cloverdale, Australia) were measured during the first session. Anthropometry measures were conducted with shoes and all heavy clothing (i.e., jackets) removed. The body mass index (BMI) was calculated using the equation kg/m^2^.

### 3.3. Umami Discrimination Task

Following the methodology outlined by previous research [10,11,12,13,14,18] to establish umami discrimination status, participants completed 24 triangle tests during each repeated taste assessment session. The 24 triangle tests were split into two blocks of 12 tests, with a five-minute break between each block. Each triangle test contained 29 mM MSG and 29 mM NaCl, to ensure that they were sodium matched. Each triangle test contained two solutions containing a 15 mL aliquot of the same stimulus (either NaCl or MSG) and one solution containing the other stimulus (either NaCl or MSG). Participants were required to taste all three solutions, from left to right, and choose the solution that they perceived to taste different from the other two solutions. Between each triangle test, participants were required to rinse their mouth out with water. Twelve tests contained two solutions of MSG and one of NaCl, and the other 12 contained two solutions of NaCl and one of MSG.

To determine participants’ umami discrimination status, the sum of the triangle test results was calculated. If participants correctly identified ≥13/24 tests, they were considered a UD, and this was further split into high discriminators (HDs) (≥18/24) and moderate discriminators (MDs) (13–17/24). Participants who correctly identified ≤12/24 were classified as NDs [12].

### 3.4. Detection Threshold and Recognition Threshold

During each repeated taste assessment session, DT and RT were measured for sweet, salty, and umami (MSG and MPG) tastes, following a modified International Standard Organisation method (ISO3972, 1991) [19], in duplicate. This was split into two blocks, with a five-minute break between. Within each block, participants’ DTs and RTs were assessed for each tastant independently, and in each block, the presentation order of the tastants was randomized. At the end of each tasting session, participants had completed DT and RT tasks for all four tastants in duplicate. Prior to testing, participants underwent familiarization tasks for each taste. Participants were provided with step number 6 (Table 1) for each taste and were informed which taste they were assessing. Participants were asked if they could perceive the presented taste. If participants could perceive the presented taste, then the taste assessment began. If not, they were presented with one step stronger until they could perceive the taste, then they could commence the taste assessment.

The taste assessment involved presenting participants with 10 concentrations of each tastant, in an ascending concentration. Each solution was presented to participants with unique 3-digit blinding codes. Participants began tasting at the lowest concentration and continued through to the highest concentration. Participants were instructed to put the entire 15 mL solution in their mouth for 5 s to taste the solution, and then, they were asked to expectorate the solution and immediately rate their taste perception. Participants recorded which taste they perceived from a list that included ‘the solution tastes like water’, ‘the solution tastes like something other than water, but I am not sure what’, ‘sweet’, ‘sour’, ‘salty’, ‘bitter’, and ‘umami’. Between each solution, participants were asked to rinse their mouth out with water. The DT was the lowest concentration at which the participants responded ‘the solution tastes like something other than water, but I am not sure what’. The RT was the lower of the two concentrations where the taste was first correctly identified twice consecutively. Thus, the lower a participant’s DT or RT, the higher their taste sensitivity.

### 3.5. Suprathreshold Intensity Perception

Participants rated the taste intensities of sweet, salty, and umami (MSG and MPG) solutions on a labelled magnitude scale (LMS), which was anchored at 0 (no sensation) to 100 (strongest imaginable) [20], whereby a higher intensity rating indicates a higher taste perception. Three test concentrations and a blank were prepared for each of the stimuli. Concentrations were low (29 mM), medium (200 mM), and high (400 mM), and the blank contained filtered water in the absence of the tastant. Concentrations were the same for all four tastants. Participants were provided with 15 mL aliquots of each solution and were instructed to taste each sample by swirling the sample in their mouth, expectorating, and then rating their taste perception on an LMS. To minimize carry-over effects, participants were asked to rinse their mouths out with water after rating each sample. Participants were provided with a tray containing one tastant at a time, with each concentration of solution presented in duplicate (excepting the blank). Thus, each tray contained seven samples. The tasting order of the samples on each tray was randomized, and the presentation order of the tastants was also randomized. A mean taste intensity rating was taken for each participant, which was the participant’s average rating of the seven samples per tastant; this was used for the LMS standardization.

### 3.6. LMS Standardization

To ensure individual difference in scale use was controlled for, participants rated the heaviness of six visually identical weights on an LMS. These weights were 600 mL bottles filled with water to make up the weights 53 g, 251 g, 499 g, 724 g, 897 g, and 1127 g and covered in aluminium foil to ensure all bottles were visually identical [21,22]. Participants rated these in ascending weight order and then repeated this in descending weight order [21,23]. For every participant, the heaviness ratings of the weights were averaged across the six weights in duplicate, producing a mean heaviness rating. Significant correlations were found between the heaviness ratings of the weights and mean taste intensity rating for salt (*r* = 0.35, *p* = 0.005), umami (MSG) (*r* = 0.41, *p* = 0.010), umami (MPG) (*r* = 0.22, *p* = 0.010), and sweet (*r* = 0.33, *p* = 0.010). As these variables should be unrelated, this indicates that standardization of the scale use was required across participants [19,24]. To determine the standardization factor, the grand mean of heaviness across the weights was divided by each participant’s individual mean intensity of the weights. Each participant’s taste intensity ratings for all tastes and concentrations were then multiplied by the participant’s individual standardization factor to eliminate scale-use bias [21,22,23].

### 3.7. Food Frequency Questionnaire

To assess habitual food intake, participants completed a food frequency questionnaire (FFQ), adapted from the 1995 Australian National Nutrition Survey FFQ [25], utilized by prior studies [26,27]. Participants indicated how many times, on average, over the previous month, they had consumed different food or beverage items. Data from the FFQ were converted into a daily occasion of consumption equivalent value, and food items were categorized into food groups based on the classification system utilized in the 2011–13 Australian Health Survey [28]. The daily occasion of consumption equivalent values were the following: ‘never or less than once a month’ = 0.02, ‘one to three times per month’ = 0.07, ‘once per week’ = 0.1, ‘two to four times per week’ = 0.4, ‘five to six times per week’ = 0.8, ‘once per day’ = 1.0, ‘two to three times per day’ = 2.5, ‘four to five times per day’ = 4.5, and ‘six plus times per day’ = 6 [27]. Nine categories were created, including fruit; vegetables; grains and cereals; seeds and nuts; meat and poultry; fish and seafood; egg products; and discretionary foods, which was further subdivided for the analysis of foods exhibiting a salty taste (i.e., salty discretionary foods).

### 3.8. Statistical Analysis

The descriptive analyses of umami discrimination status, taste assessment measures, and demographics are presented as the mean ± SD, except for sex, which is reported as *n* (%). Statistical significance was set at *p* < 0.05 for all analyses, and analyses were conducted using IBM SPSS statistical software version 28.0 (Armonk, NY, USA: IBM Corp).

To determine whether there were differences in sex between discrimination groups, a Fisher’s exact test for independence was conducted. To determine whether any differences in BMI and age existed between the discrimination categories and also the sex categories, Kruskal–Wallis tests were conducted.

The distributions of all variables were visually observed for normality. Salty, umami (MSG and MPG), and sweet ST variables were positively skewed, requiring a logarithmic transformation to approximate normal distribution. Associations (β) between discrimination status and taste assessment (i.e., salt RTs) were assessed using linear mixed-effects models. The models included the taste rating as a fixed effect and the participant’s unique study ID as a random effect to account for all the repeated measures obtained by each participant. We included age as a covariate to account for any effect of age on taste; BMI was also assessed in the models but was only associated with ST for umami tastants (MSG and MPG) and was thus only used in the ST MSG and MPG models. A *post hoc* Sidak’s test was used to compare means between the discrimination status and to correct for multiple comparisons. We report the pairwise comparison of the between-group differences, 95% CI, and *p*-values.

A univariate ANOVA was used to examine the effects of discrimination status on food frequency consumption of the various food groups. Sex was included as a covariate to account for the difference in food intake between males and females. As participants attended different numbers of repeated taste assessment sessions, the model was weighted to adjust for the number of sessions each participant attended to ensure that we accounted for the repeated measures obtained. A *post hoc* Sidak’s test was used to compare the means between discrimination statuses and to correct for multiple comparisons; we report 95% CI and *p*-values.

## 4. Results

### 4.1. Taste Measures, Umami Discrimination Status, and Demographics

In the analysis, *n* = 61 subjects were included, with *n =* 40 females and *n =* 21 males, a mean age of 29.6 ± 8.3 years, and a mean BMI (kg/m^2^) of 26.1 ± 6.7. A total of *n* = 9 participants (15%) were umami NDs, whereas *n* = 52 (85%) were UDs, of which *n* = 29 (48%) were HDs. There were no significant differences in the proportions of UDs and NDs and HDs, MDs, or NDs between males and females (*p* > 0.05). There were no significant differences in the age or BMI between any the discrimination groups nor between males and females (all *p* > 0.05). The mean DT (step), RT (step), and ST ratings, overall and split by discrimination status, are presented in Table 2.

### 4.2. Effect of BMI on Taste Measures

A higher BMI was associated with a lower intensity perception of MSG (β: −0.01, *p* = 0.03), and it was trending towards significant for MPG (β: −0.01, *p* = 0.06). No associations were observed between BMI and the DT or RT of any tastants or the ST of sucrose and salt (*p* > 0.05). Based on these interactions, BMI was included as a covariate in the final linear mixed effects models for the MSG and MPG ST ratings.

### 4.3. Comparison of DT between Discriminator Status

There were no statistically significant differences between discrimination statuses (UD and ND) for the salt (*p* = 0.67), MSG (*p* = 0.43), MPG (*p* = 0.96), or sucrose (*p* = 0.90) DTs. Similarly, there were no statistically significant differences between discrimination statuses (HD, MD, and ND) and the salt (*p* = 0.76), MSG (*p* = 0.40), MPG (*p* = 0.77), or sucrose (*p* = 0.85) DTs.

### 4.4. Comparison of RT between Discriminator Status

UDs had lower salt (mean difference (MD): −1.58 steps [95% CI: −3.08, −0.29], *p* = 0.03), and sucrose RTs (MD: −1.34 steps [95% CI: −2.54, −0.15], *p* = 0.03) than NDs. There were no statistically significant differences between discrimination status (UD vs. ND) and MSG (*p* = 0.31) or MPG (*p* = 0.41) RTs. When looking further at the split of HD and MD and ND subgroups, there were no statistically significant differences between salt (*p* = 0.06), MSG (*p* = 0.26), MPG (*p* = 0.14), or sweet (*p* = 0.093) RTs.

### 4.5. Comparison of ST Ratings between Discriminator Status

UDs rated MPG as more intense than NDs (MD: 0.21 [95% CI: 0.17, 0.40), *p* = 0.03), and when participants were split into HDs, MDs, and NDs, MDs rated the MPG ST samples as more intense than NDs (MD: 0.27 [95% CI: 0.01, 0.53], *p* = 0.04). There was no difference in the salt (*p* = 0.13), MSG (*p* = 0.27), or sweet (*p* = 0.32) RTs between UDs and NDs nor when split into HDs, MDs, and NDs (all *p* > 0.05).

### 4.6. Comparison of Habitual Food Intake between Discriminator Status

**Figure 2:**
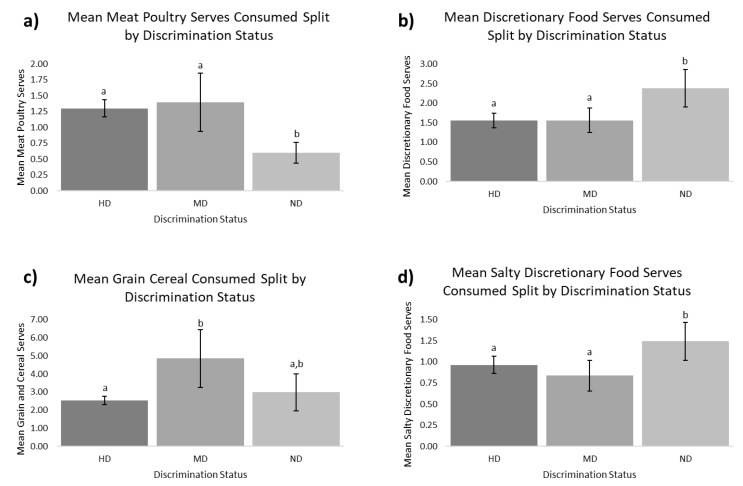
Mean reported serves of daily intake by discriminator status. (**a**) Mean meat and poultry serves; (**b**) mean discretionary food serves; (**c**) mean grain and cereal serves; (**d**) mean salty discretionary food serves reported in the FFQs. Different letters represent significant differences (*p* < 0.05) following Sidak’s test for multiple comparisons between groups; 95% CI error bars are presented. The discrimination status is presented as umami discriminators (UDs) (≥13/24 correct), *n* = 52, and is further split into high-discriminators (HDs) (≥18/24 correct), *n* = 29; moderate discriminators (MDs) (13–17/24 correct), *n* = 23; and non-discriminators (NDs) (8–12/24 correct), *n* = 9.

We found a main effect of discrimination status on the mean daily serves of discretionary food (F(2,168) = 6.77; *p* = 0.001), meat and poultry (F(2,168) = 4.04; *p* = 0.02), salty discretionary food (F(2,168) = 5.63; *p* = 0.004), and grain and cereals (F(2,168) = 6.99; *p* = 0.001), as shown in Figure 2. There was no observed effect of discrimination status on the consumption of vegetables (*p* = 0.81), fruit (*p* = 0.86), fish and seafood (*p* = 0.12), seeds and nuts (*p* = 0.83), or egg products (*p* = 0.54).

## 5. Discussion

The discrimination status method was successful in classifying the sample population into NDs and UDs. In this study, the ND sub-population consumed less meat and poultry, suggesting a role of glutamate discrimination in protein-rich meat consumption. This study also observed that NDs were less sensitive to salt at RT and consumed more discretionary and, specifically, salty discretionary food. Whilst NDs make up a minority (15%) of this study population, the reduction in salt RT, the increased consumption of salty discretionary food, and the reduced consumption of meat and poultry makes the NDs an important sub-population for future research.

### 5.1. Umami Discrimination and Taste

At RT, NDs were less sensitive to salt taste than UDs. It appears that the discrimination task provides information regarding sodium sensitivity and that sodium is the dominating ion for discrimination capabilities. Considering that the discrimination task not only involves MSG but also NaCl, it is logical that salt RT are associated with an individual’s ability to discriminate between the two solutions. Supporting this, umami-sensitive individuals (measured through RTs) had stronger neural responses in primary gustatory areas to both salty and umami stimuli compared to umami-insensitive individuals [29]. Different central processing between UDs and NDs feasibly plays a role in both umami and salty perception [29]. This is supported in psychophysical studies where umami–salty taste confusions are evident [30], and NDs’ salt and MSG DTs were highly correlated (r = 0.75) but not for UDs (r = 0.25) [10]. We add to this by demonstrating that NDs exhibit reduced salt RTs in comparison to UDs, from which we conclude that sensitivity to the Na+ ion contributes to discrimination capabilities.

The results illustrate the complexity of measuring the taste perception of L-glutamate in isolation, as sensitivity to the cation (i.e., Na+ or K+) will influence results. NDs also exhibited a somewhat reduced RT to MSG compared to UDs (6.6 vs. 7.5 steps). Their reduced perception of MSG is conceivably a result of reduced Na+ sensitivity, but reduced sensitivity to L-glutamate likely also contributes to some extent, as we observed a lower MPG RT, particularly for HDs compared to NDs (6.1 vs. 7.3 steps). Nevertheless, we do not know what impact the K+ cation has on the taste perception of MPG and whether this contributes to differences in taste perception between discrimination groups. A study found a significant reduction in MSG RTs in NDs; however, MPG and salt RTs were not assessed [11]. Perhaps, the degree of dissociation of the salts contribute to NDs’ reduced ability to discriminate between MSG and NaCl, with MSG having a reduced degree of disassociation compared to NaCl in solution [9]. NDs are less sensitive to Na+ in NaCl, so they conceivably perceive reduced input from the Na+ ion in MSG and have a reduced perception of L-glutamate due to their inability to discriminate NaCl and MSG. Potentially, the combined contribution of the Na+ and L-glutamate in MSG may, for the NDs, result in a similar taste perception to NaCl when presented at isomolarity. Finally, UDs had lower sucrose RTs than NDs. Prior research has similarly shown that umami hypotasters have a reduced sweet RT [15]. This suggests potential involvement of the T1R3 G-protein-coupled receptor, considering that sweet and umami compounds both utilize this receptor [2,16,31]. However, this is speculative, and further research is required to determine the involvement of the shared receptor dimer in sweet and umami taste associations, particularly as we observed no significant differences in MSG and MPG RTs.

Following from this, the only finding observed at ST was the reduction in the MPG intensity for NDs compared to UDs (HDs and MDs). Chen and colleagues similarly found that NDs rated the intensity of MPG lower in comparison to UDs and also did not observe a difference in the sweet taste intensity [11]. Conversely, one study demonstrated that umami NDs perceive lower sweet intensity with sucrose and lower savoriness intensity with MSG but have a higher saltiness perception of MSG [13]. Conversely, another study demonstrated that umami NDs perceive salt intensity lower than UDs [14]. Notably, umami taste dimensions have not been shown to covary [10,19]; thus, the discrimination task may not be useful for predicting ST intensity perception. It is important to note that although intensity ratings from this study and prior research were all measured using the LMS, perceived intensity is a function of the concentrations used (i.e., the maximum sucrose concentration varied from 400 mM [11] to 1 M [13]) and tastant used for each taste (i.e., MSG [13] or MPG [11]). Therefore, contrasting findings between studies could be attributed to this, and consistent concentrations and tastants across studies are required for accurate comparisons. While some specific differences in taste perception across both RTs and STs were observed in NDs, it is important to note that the descriptive statistics display that, overall, the NDs were less sensitive across all four tastes for STs and RTs. Potentially, the discrimination task isolates a group of participants with an overall weakened taste sensitivity.

### 5.2. Umami Discrimination and Dietary Intake

We observed that the 29 mM discrimination task provided information related to the dietary intake of protein-rich meat and poultry and salty-dominant foods. NDs consumed less meat and poultry and more discretionary and, specifically, salty discretionary food than UDs. The mechanism for this association is not understood. Potentially, an increased ability to sense protein in food acts as a sub-conscious accelerator of consumption for UDs. This is reported in other alimentary tastes, such as carbohydrate taste, whereby increased sensitivity is associated with increased consumption of carbohydrate-rich foods [32]. A hedonic response may also be involved in the increased protein consumption, although prior research is mixed for umami. Increased umami taste sensitivity is associated with a greater preference for high-protein foods in healthy individuals [33], a higher intake of seaweeds but not meat and poultry [15], and a lower consumption of salty-savory foods [34]. One study has shown that increased consumption of MSG over a month led to increased liking and wanting of high-protein foods but a decreased appetite for savory food [35]. Notably, the savory food included in the aforementioned study was pasta, which is not a typical high-protein meal. Moreover, after the month-long intervention, MSG ST taste intensity decreased in the female participants following increased MSG consumption [35]. From the present study, we cannot ascertain causality (i.e., dietary intake influencing taste perception or taste perception influencing dietary intake), but prior research suggests that MSG consumption may impact MSG taste perception. While further research is required, it may be that the importance of discrimination status in terms of food liking and dietary intake is related to both high-protein content in foods and, presumably, the ability to detect this protein and the presence of L-glutamate.

Discretionary food consumption is nutritionally relevant due to excessive salt intake. NDs had a significantly lower salt RT than UDs, suggesting that a reduced ability to recognize salt is associated with an increased consumption of energy-dense and sodium-rich food. As sodium is an essential nutrient, the ability to regulate salt intake may be linked in some part to the ability to recognize salt in food at lower concentrations and, thus, leading to salt-specific satiety. Some studies have failed to show an association between salt RT and dietary intake [36,37,38,39]. Other studies have found that a higher salt RT is associated with increased energy intake [40], increased salty and high-energy food intakes [41], the addition of salt to food prior to tasting [42], and increased salt intake [40,43]. Adding to the prior research, we found that NDs were less sensitive to salt and reportedly consumed more discretionary food and salty discretionary food than UDs. Finally, we observed significant differences in grain and cereal intake between HDs and MDs, but it is unclear what the mechanisms for this are. Grain and cereal products contribute significantly to dietary sodium consumption in Western society [44] whilst not tasting intensely salty [45]. Perhaps, we may have similarly anticipated an increased consumption of these foods in NDs; however, only the MDs consumed significantly more than the HDs. The discrimination task may be more relevant to the consumption of salt than the traditional assessment of DT, RT, and ST. Therefore, we suggest that the discrimination status method provides important information about both sodium and umami discrimination and associated food intake. Based on these findings, the umami discrimination tool may be more appropriately termed the glutamate–sodium discrimination tool.

### 5.3. Umami Taste and BMI

This study adds to prior research suggesting that umami taste perception may be attenuated in participants with a higher BMI; however, findings are not always consistent across different taste dimensions [13]. Higher intensity ratings were associated with lower BMIs at ST for MSG and MPG, but this was not reflected in the DT and RT results. A prior study has similarly reported that reduced intensity perception of umami is associated with a higher BMI [34]. At umami RT, lean individuals (BMI < 25.0 kg/m^2^) have been found to have an increased sensitivity compared to overweight subjects (BMI 25.0–29.9 kg/m^2^) [30]. However, another study observed mixed results, with obese women preferring high concentrations of MSG in food and having a reduced sensitivity at DT but no difference in the umami ST intensity perception compared to normal-weight subjects [13]. The strength of the findings in the present study is highlighted by the association between increasing BMI and the reduced ST intensity of both L-glutamate-containing stimuli (MSG and MPG).

### 5.4. Limitations

It is important to recognize some limitations of the present study. Inconsistent findings with previous research for STs are likely to be a function of variations in concentrations and tastants used, both in the present study and in prior research, making it difficult for comparisons to be made. It is also important to note that, conceivably, the NDs have a weakened overall taste perception, as they had lower RTs and STs across all four tastes. Further research investigating other taste perceptions (i.e., sour and fat tastes) in these NDs would be needed to ascertain whether they do have an overall taste insensitivity. While the study included 61 participants, the ND group was 15% of the sample population, making them a minority subgroup of the population. However, considering that we observed significant findings between NDs and UDs with the current sample sizes, this suggests that the findings are important. Finally, this is a cross-sectional study, and it only illustrates associations rather than causality.

## 6. Conclusions

This study has been the first to evaluate differences in taste perception between UDs and NDs, using both MSG and a non-sodium umami stimuli MPG, across three taste dimensions (DT, RT, and ST). We showed that NDs are less sensitive to salt and sucrose RTs, but surprisingly, they are not for MSG and MPG RTs. This may be due to Na+ being the dominant ion for NDs, and glutamate being less so. Potentially, the discrimination task provides more information about sodium discrimination than L-glutamate discrimination. We suggest that the discrimination tool should be more appropriately termed the glutamate–sodium discrimination tool. The discrimination status provides us with insights into NDs’ taste and dietary intake. Being able to identify these NDs may provide insight into a population at risk of the overconsumption of discretionary food and reduced intake of protein. Further research should investigate the acute and habitual dietary intake of these NDs and associations with RTs in a larger study. Moreover, understanding the characteristics (i.e., genetics, saliva, receptor expression) of these NDs to ascertain why these participants have a reduced ability to discriminate between sodium and L-glutamate is required.

## Figures and Tables

**Table 1:** Tastes and tastants used in taste assessment and the respective sample concentration steps (1–10) and concentrations (mM).

Taste	Tastant		Sample Concentrations (mM)									
		Step:	1	2	3	4	5	6	7	8	9	10
Sweet	Sucrose		1	1.6	2.7	4.5	7.5	12.6	21.0	35.0	70.0	140
Salty	NaCl		2.4	4.1	5.8	8.2	11.8	16.8	24.0	34.2	68.4	137
Umami	MSG		0.5	0.7	1.0	1.4	2.0	2.9	4.1	5.9	11.8	23.6
Umami	MPG		0.5	0.7	1.0	1.4	2.0	2.9	4.1	5.9	11.8	23.6

**Table 2:** Mean taste dimension measures (DT, RT, and ST) for salt, MSG, MPG, and sucrose assessments, split by discrimination status ^1^.

Taste Measure	Discrimination Status	Salt	MSG	MPG	Sucrose
DT (step)	All participants ^2^	1.97 ± 1.57	1.95 ± 1.64	2.01 ± 1.63	2.30 ± 1.94
	UD ^3^	1.98 ± 0.16	2.02 ± 0.18	1.99 ± 0.16	2.28 ± 0.20
	HD ^3^	1.87 ± 0.21	1.83 ± 0.24	1.86 ± 0.22	2.33 ± 0.26
	MD ^3^	2.14 ± 0.24	2.27 ± 0.27	2.16 ± 0.25	2.21 ± 0.30
	ND ^3^	1.99 ± 0.39	1.45 ± 0.44	1.76 ± 0.40	2.08 ± 0.49
RT (step)	All participants ^2^	5.34 ± 2.69	6.53 ± 3.22	6.57 ± 3.08	6.04 ± 2.52
	UD ^3^	5.27 ± 0.26	6.59 ± 0.32	6.61 ± 0.31	5.90 ± 0.22
	HD ^3^	5.09 ± 0.34	6.24 ± 0.42	6.11 ± 0.39	5.92 ± 0.29
	MD ^3^	5.52 ± 0.39	7.07 ± 0.484	7.28 ± 0.46	5.87 ± 0.35
	ND ^3^	6.84 ± 0.64	7.47 ± 0.78	7.28 ± 0.74	7.24 ± 0.56
ST Ratings (log transformed)	All participants ^2^	1.55 ± 0.27	1.41 ± 0.28	1.44 ± 0.31	1.40 ± 0.27
	UD ^3^	1.56 ± 0.03	1.43 ± 0.33	1.47 ± 0.04	1.41 ± 0.04
	HD ^3^	1.52 ± 0.05	1.38 ± 0.04	1.43 ± 0.05	1.35 ± 0.05
	MD ^3^	1.61 ± 0.05	1.49 ± 0.05	1.53 ± 0.06	1.48 ± 0.05
	ND ^3^	1.43 ± 0.82	1.33 ± 0.08	1.26 ± 0.09	1.32 ± 0.09

^1^ Discrimination status is presented as umami discriminators (UDs) (≥13/24 correct), *n* = 52, further split into high-discriminators (HDs) (≥18/24 correct), *n* = 29; moderate discriminators (MDs) (13–17/24 correct), *n* = 23; and non-discriminators (NDs) (8–12/24 correct), *n* = 9. ^2^ The unadjusted mean ± SD are presented. ^3^ The model-adjusted mean ± SEM presented.

## Data Availability

Data described in the manuscript, code book, and analytic code will be made available upon request, pending due to ethics; please make a request to the CASS Food Research Centre, at Deakin University: cass@deakin.edu.au.
